# Efficacy of Cefiderocol Against Endophthalmitis Isolates

**DOI:** 10.3390/antibiotics13121236

**Published:** 2024-12-23

**Authors:** Brennan Schilling, Michael Hii, Hazel Q. Shanks, Eric G. Romanowski, Jonathan B. Mandell, Robert M. Q. Shanks, Michael Zegans

**Affiliations:** 1 Dartmouth-Hitchcock Medical Center, 1 Medical Center Drive, Lebanon, NH 03756, USA; brennan.m.schilling@hitchcock.org (B.S.);; 2 The Charles T. Campbell Ophthalmic Microbiology Laboratory, Department of Ophthalmology, University of Pittsburgh School of Medicine, 1622 Locust St., Pittsburgh, PA 15219, USA

**Keywords:** endophthalmitis, cefiderocol, antibiotic resistance, Gram negative

## Abstract

**Background/Objectives:** Endophthalmitis is an intraocular microbial infection that can lead to permanent blindness, even with prompt anti-microbial therapy. Multi-drug-resistant organisms are on the rise, potentially limiting the efficacy of current empiric antibiotic therapies of intravitreal ceftazidime and vancomycin. Cefiderocol is a recent FDA- and EMA-approved antibiotic for multi-drug-resistant Gram-negative bacteria. **Methods:** To better understand its potential utility in the treatment of ocular infections, the MIC of cefiderocol was compared to ceftazidime and amikacin in endophthalmitis bacterial isolates using Epsilometer testing. Because vancomycin is commonly given concomitantly as part of empiric endophthalmitis treatment, possible synergistic and antagonistic effects of concomitant vancomycin and cefiderocol were also evaluated. **Results:** Cefiderocol was found to have lower MIC values compared to ceftazidime for *Pseudomonadales* or *Enterobacterales* species. When comparing the MICs of cefiderocol and vancomycin, there appeared to be no antagonism between the two antibiotics. **Conclusions:** This is the first report exploring the use of cefiderocol in endophthalmitis strains. The results of this study show this is a promising antibiotic for multi-drug-resistant Gram-negative organisms but further research is needed to investigate its intraocular safety profile.

## 1. Introduction

Endophthalmitis is a rapidly progressive vision-threatening infection. It is a rare but feared complication of any intraocular surgery. While a recent study of 9,744,400 intraocular surgeries among Medicare beneficiaries from 2016 to 2019 found an overall rate of 0.09%, this varied significantly for different procedures. The authors reported that endophthalmitis occurred in 0.43% of corneal transplants, 0.36% of secondary intraocular lens (IOL), which are placed secondary to a variety of complications associated with the patient’s first cataract surgery, 0.24% of retina procedures, 0.16% of glaucoma procedures, 0.11% for cataracts combined with other procedures, and 0.08% of cataract surgeries [1]. These variations likely reflect differences in the duration, complexity, fluid exchange, and the post-operative integrity of surgical wounds among these different procedures.

Endophthalmitis can be due to both endogenous and exogenous causes. Endogenous endophthalmitis itself is commonly associated with systemic bloodstream infections and the hematogenous seeding of pathogens to the eye often via circulation to the choroid, which is the layer of blood vessels and connective tissue between the white of the eye (sclera) and the retina at the back of the eye. This leads to a posterior-to-anterior development of endophthalmitis. Interestingly, the right eye seems to be more commonly impacted in endogenous endophthalmitis due to more direct flow from the right carotid artery [2]. It is also important to note that studies have shown up to 44% of cases occurred without an identifiable infectious source, but pseudomonas and fungi are more commonly implicated compared to exogenous endophthalmitis [3]. Immunosuppression and indwelling catheters can contribute to the cause of endogenous endophthalmitis without an apparent infectious source. Exogenous endophthalmitis more commonly occurs following the introduction of pathogens into the eye from an outside source such as intraocular surgery, intravitreal injections, or trauma. Post-traumatic endophthalmitis can be due to a variety of pathogens, but it has been linked with *Staphylococcal* and *Bacillus* species [4]. In such cases, the inoculum may come from the environment or from the patients’ microbial flora.

Defining the subsets of post-operative exogenous endophthalmitis is useful in identifying the most common pathogens. In acute post-operative endophthalmitis, defined as within six weeks of surgery, infection with the normal flora of the surrounding ocular structures is most common [5]. *Staphylococcal epidermidis* is the bacterial species most commonly isolated from these cases [5]. Delayed/chronic endophthalmitis is defined as 6 weeks after surgery, and this continues to have a bacterial predominance, in particular, *Cutibacterium acnes*, but fungal etiologies start to increase in likelihood. Cataract surgery is the most common intraocular surgery and, accordingly, most cases of post-operative endophthalmitis that occur are associated with it. However, other intraocular surgeries have different patterns of post-operative endophthalmitis. Delayed cases are often associated with bleb-related surgeries, with *Streptococci* and *Haemophilus* tending to be the common microbes [6].

Patients typically present with acute eye pain, redness, vision change, and headache. Failure to properly identify endophthalmitis can lead to very poor visual outcomes and possible evisceration [7]. Because of this urgency to treat to save visual potential, empiric treatment with intravitreal antibiotics is often started prior to the identification of the causative organism and determination of its antibiotic susceptibilities. Case series have been performed, which have revealed that Gram-positive organisms are more often identified as the causative organism than Gram-negative organisms, but there is still a necessity to provide Gram-negative coverage [8,9]. The most common bacterial pathogens implicated in a 25-year single center review of endophthalmitis isolates are as follows: *Staphylococcus epidermidis* (30.3%), other coagulase-negative *Staphylococcus* (9.1%), *Streptococcus viridans* (12.1%), *Staphylococcus aureus* (11.1%), *Enterobacteriaceae* (3.4%), and *Pseudomonas aeruginosa* (2.5%) [10].

The pathophysiology of endophthalmitis is secondary to breakdown of the blood aqueous barrier or the blood retinal barrier via a variety of mechanisms, like trauma or surgical intervention. Pathogens are then able to colonize the interior of the eye [5]. In the past, susceptibility analysis revealed that 100% of tested Gram-positive organisms were susceptible to vancomycin and 100% of tested Gram-negative organisms were susceptible to ceftazidime [8]. Aminoglycosides have also been considered for treatment, though less commonly due to possible toxic effects [8]. In the setting of studies like these, the classic antibiotics of choice have been vancomycin for Gram-positive coverage and ceftazidime for Gram-negative coverage. Antifungal agents would be added if there are risk factors for fungal etiology. However, this historical standard of care is likely to be flawed in the setting of new drug-resistant organisms. In a large and more recent series, 8% of cases secondary to Gram-negative bacteria were resistant to ceftazidime [11]. Reflective of the virulence of Gram-negative endophthalmitis, the authors also reported that 30% of affected patients required enucleation or evisceration [11].

This problem has been highlighted by the recent outbreak of ocular infection with carbapenem-resistant *Pseudomonas aeruginosa* (PA) with Verona integron-mediated metallo-β-lactamase and Guiana extended-spectrum-β-lactamase (VIM-GES-CRPA) associated with EzriCare artificial tears [12,13]. This specific strain of PA was found to be only susceptible to cefiderocol. Antibiotic susceptibility testing of the outbreak strain by the Centers for Disease Control and Prevention (CDC) produced minimum inhibitory concentrations (MICs) for the antibiotics used to commonly treat endophthalmitis, ceftazidime (>128 µg/mL), and amikacin (>64 µg/mL) that were highly resistant to those antibiotics but susceptible to cefiderocol (0.5 µg/mL) [14]. A recent systematic review investigating the utility of cefiderocol in 2022 revealed promising results with effective in vitro and in vivo activity against many multi-drug-resistant Gram-negative organisms; however, it was only approved by the FDA in 2020 for complicated urinary tract infections and nosocomial pneumonia [15]. Cefiderocol has recently been shown to be effective in vitro and in vivo for the topical treatment of PA keratitis in a rabbit model [13]. Given these promising results, further investigation is warranted to identify the utility of cefiderocol for Gram-negative coverage in global empiric treatment of endophthalmitis. As a first step to understanding the utility of cefiderocol in endophthalmitis, the antibiotic was tested in vitro against both Gram-positive and Gram-negative endophthalmitis isolates from eastern North America.

## 2. Results

Table 1 presents the 50% and 90% minimum inhibitory concentrations (MIC_50_ and MIC_90_) for both Gram-negative and Gram-positive endophthalmitis isolates. These are the antibiotic concentrations that inhibit the growth of 50% and 90% of the bacterial isolates tested in a given set. *Pseudomonadales*, *Enterobacterales*, and other Gram-negative species were found to have lower MIC_50_ and MIC_90_ values to cefiderocol than amikacin or ceftazidime. Cefiderocol was similarly effective based on MIC_50_ and MIC_90_ compared to the other two antibiotics with regard to other Gram-negative species, but as expected, the Gram-positive isolates of *Staphylococcus aureus* and coagulase-negative *Staphylococcus* species (CNS) had greater MIC_50_ and MIC_90_ values compared to cefiderocol (Table 1).

The MIC data were used to determine susceptibilities of the isolates to cefiderocol, amikacin, and ceftazidime. CLSI susceptibility breakpoints are antibiotic concentrations achieved after systemic administration of the antibiotics based on successful outcomes of treatments. These systemic breakpoints are used as guidelines for topical and intravitreal therapy, as no susceptibility standards exist for these therapies. Based on CLSI susceptibility breakpoints, there were no significant differences in susceptibility for *Pseudomonadales* (Table 2). This lack of difference may be secondary to the use of systemic CLSI susceptibility breakpoints. In contrast, the *Enterobacterales* isolates were significantly more susceptible to cefiderocol and ceftazidime than amikacin (Fisher’s exact test, *p* ≤ 0.0205) (Table 2). Therefore, the use of amikacin may not be the best antibiotic for the empiric therapy of endophthalmitis compared to ceftazidime or possibly cefiderocol.

The interaction between cefiderocol and vancomycin on antimicrobial activity was also investigated. Synergy or antagonism was evaluated and the FIC indices for each tested bacterium were between 1.0 and 4.0, which are considered indifferent, which in this case means there is no interaction between cefiderocol and vancomycin (Table 3).

## 3. Discussion

Cefiderocol is a synthetic antibiotic consisting of a cephalosporin group and a siderophore group. Cefiderocol’s siderophore group binds to iron in the environment. The iron containing antibiotics is then transported into the bacterial cell via active iron transporters. In the bacterial periplasmic space, the siderophore group is removed and the ceftazidime-like cephalosporin group binds to penicillin-binding protein 3. The antibiotic then in turn inhibits bacterial cell wall synthesis [16]. The use of iron-bound siderophore group tricks the bacteria into taking up the iron-containing antibiotic. Because of this, cefiderocol is considered a “Trojan Horse” antibiotic [17].

The results of this study suggest cefiderocol may have increased efficacy when treating ocular *Pseudomonadales* or *Enterobacterales* infections compared to the commonly used amikacin or ceftazidime. This suggests that its use is most promising with specific drug-resistant Gram-negative bacteria and further supports its use in multi-drug-resistant infections. It is also important to note the effect of iron on the function of cefiderocol. Cefiderocol relies on iron uptake systems to function well, specifically in low iron concentrations. Iron concentration is specifically low in infected tissues, increasing its efficacy [18,19]. The fact that iron levels at baseline in the vitreous is lower than plasma suggests that the efficacy of cefiderocol may be greater in the eye than prior research investigations in its utility for UTIs and nosocomial pneumonia.

Typically, endophthalmitis is empirically treated with a combination of two antibiotics, one to cover Gram-positive bacteria and the second to cover Gram-negative bacteria. Currently vancomycin is used to cover potential Gram-positive pathogens, while ceftazidime or amikacin are used for potential Gram-negative pathogens. Typically, endophthalmitis cases are caused by Gram-positive bacteria. For example, at the Charles T. Campbell Opthalmic Microbiology Laboratory at the University of Pittsburgh, 93% of bacterial endophthalmitis pathogens are Gram-positive and 7% are Gram-negative [20]. As determined in the current study, there was resistance to ceftazidime and amikacin among the *Enterobacterales* isolates, but no isolates were resistant to cefiderocol. Therefore, cefiderocol could be considered as a more potent alternative to ceftazidime and amikacin for Gram-negative coverage. Cefiderocol has little to no activity against Gram-positive cocci [21], the most common Gram-positive endophthalmitis pathogens isolated [20]. Therefore, cefiderocol would be used exclusively to cover Gram-negative pathogens.

Encouragingly, we did not observe any in vitro antimicrobial antagonism between cefiderocol and vancomycin when used concomitantly and, in fact, we observed lower MICs with this combination. The intravitreal safety profile for cefiderocol may be similar to that of ceftazidime based on structural similarities [22], but given the well-documented retinal toxicity of some antibiotics (aminoglycosides) [23], studies to investigate the intravitreal safety of cefiderocol are necessary, since the effects of intraocular cefiderocol are unknown. Furthermore, the concentration of intraocular cefiderocol, as well as the pharmacokinetics of intraocular injections of cefiderocol, must be determined. Hemorrhagic occlusive retinal vasculitis (HORV) is a rare but significant complication of using intraocular vancomycin. It typically has delayed onset, with findings including retinal hemorrhage, retinal nonperfusion, and vascular sheathing [24]. This is further highlighted by the recent similar retinal findings associated with intravitreal peptidoglycan for dry age-related macular degeneration. Studies in 2024 have shown associations with these injections and retinal vasculitis [25]. Taken together, these complications associated with other intraocular medications point to the importance of investigations of the potential retinal toxicity of cefiderocol. The first step toward analyzing the safety profile of cefiderocol will be to perform in vitro toxicity assays against retinal cells.

Cases of Gram-negative bacteria with either baseline or other forms of antibiotic resistance to cefiderocol have been described. [26]. Understanding the mechanism behind the development of this resistance is vital to guide future research in treating these multi-drug-resistant organisms. Unfortunately, it appears there is not a consistent mechanism in bacterial resistance development. In one study evaluating the cause of cefiderocol resistance of carbapenem-resistant *Enterobacterales*, it appeared the two main mechanisms of resistance are beta-lactamase production and permeability defects, both common causes of carbapenem resistance [27]. Despite these caveats, it is likely that cefiderocol has a broader spectrum of activity against resistant Gram-negative bacteria, with potential therapeutic benefits.

Limitations of this study include the regional nature of isolates from the northeastern United States of America, bias toward the major endophthalmitis pathogens seen in our region, and an oversampling of Gram-negative bacteria compared to Gram-positive bacteria. These isolates, especially Gram-negative isolates, are rare and are not banked by many laboratories. Furthermore, the emphasis for the use of Gram-negative bacteria in this study is due to cefiderocol being predominantly effective against Gram-negative bacteria, with little to no activity against Gram-positive bacteria [21]. Also, it is important to recognize that there are no standardized CLSI breakpoint susceptibilities for intravitreal therapy. Studies with the goal of establishing a standardized set of breakpoints would be a worthwhile endeavor. However, utilizing these systemic breakpoints is still a reasonable way to compare any significant differences between amikacin, cefiderocol, and ceftazidime. An additional limitation of this study is the lack of genetic analyses of the bacterial pathogens used in this study to determine mechanisms of resistance to the antibiotics tested. While there was resistance to ceftazidime and amikacin demonstrated for *Enterobacterales*, there was no resistance demonstrated among the *Pseudomonadales*. Most importantly, there was no resistance demonstrated for either Gram-negative bacterial family to cefiderocol. The lack of cefiderocol resistance of Gram-negative endophthalmitis pathogens may provide ophthalmologists with an antibiotic that will cover all Gram-negative species similar to vancomycin for Gram-positive species.

Future studies are indicated to determine mechanisms of cefiderocol resistance in Gram-negative endophthalmitis if and when they are encountered. Nevertheless, this study serves to assess cefiderocol as a potential Gram-negative bacteria-specific antibiotic for endophthalmitis. However, safety trials are necessary prior to use in the clinic. As stated briefly above, we do not recommend that clinical practice be changed prior to further study to validate the use of cefiderocol in vivo.

This is the first report to explore cefiderocol susceptibility among endophthalmitis strains. Though Gram-negative endophthalmitis is less common than Gram-positive endophthalmitis, it is typically more severe, so empiric treatment always covers for both Gram-positive and Gram-negative organisms. It is more likely to be encountered in association with sepsis, trauma, Gram-negative keratitis, liver abscesses, and bleb infection. Cefiderocol provides a possible avenue to treat endophthalmitis secondary to Gram-negative bacteria in the setting of increased cases of multi-drug-resistant strains and warrants additional study, particular of its intraocular safety.

## 4. Materials and Methods

### 4.1. Bacterial Strains

Deidentified bacterial strains isolated from patients with endophthalmitis presenting to the Charles T. Campbell Ophthalmic Microbiology Laboratory at the Department of Ophthalmology at the University of Pittsburgh School of Medicine, in Pittsburgh, PA, USA, were used for in vitro minimum inhibitory concentration (MIC) determinations. Bacteria were isolated from aqueous or vitreous humor taps or total vitrectomy samples in a College of American Pathologists-certified laboratory using standard microbiological isolation techniques on trypticase soy agar with 5% sheep’s erythrocytes, chocolate agar, and mannitol salt agar and incubated at 37 °C in 6% CO_2_ for up to 5 days until colonies formed. Standard biochemical methods, *sodA* sequencing [28], or Maldi-Tof mass spectroscopy were used to identify the bacteria. Following identification and antibiotic susceptibility testing for patient treatment, the bacterial isolates were frozen at −80 °C in 15% glycerol (final volume).

The bacteria evaluated in this study were the Gram-negative orders *Pseudomonadales* (including 14 *Pseudomonas* species and 2 *Moraxella* species), *Enterobacterales* (including 10 *Serratia* species, 3 *Proteus*, *Morganella*, and *Klebsiella* species, 1 *Escherichia coli*, and 1 *Pantoea agglomerans*), and other Gram-negative bacteria including 2 *Stenotrophomonas maltophilia* and 1 *Ralstonia pickettii*. Gram-positive bacteria evaluated were *Staphylococcus aureus* and coagulase negative staphylococci (CNS).

### 4.2. MIC Testing

MIC values were determined by Epsilometer testing (E-test), as previously described [29]. Briefly, the bacterial endophthalmitis isolates were thawed and grown on trypticase soy agar with 5% sheep erythrocytes, were suspended in saline, adjusted to 0.5 McFarland standard (~1.2 × 10^8^ CFU/mL), and swabbed onto Mueller–Hinton II agar plates to form a lawn of bacteria across the entire surface of the plates. After the inoculum had dried, antibiotic strips (LIOFILCHEM, Waltham, MA, USA) were applied to the lawns gradient-label side up and incubated for 24h at 37 °C. The antibiotics tested were amikacin (cat. no. 92018), cefiderocol (cat. no. 92067), ceftazidime (cat. no. 92138), and vancomycin (cat. no. 92057). The MIC values were determined following the manufacturer’s guidelines at the point at which bacterial growth touched the strip. The MIC tests were performed at least twice for each strain, and the higher value was recorded if there was a discrepancy, as this ensures there would not be an under-estimation of the concentration of antibiotic needed for bacterial eradication. Susceptibility of the bacteria to the antibiotics was based on the CLSI systemic breakpoints. See Table 2 for the CLSI breakpoints for each antibiotic. There are no susceptibility breakpoints for the topical or intravitreal therapy of the eye.

### 4.3. Interaction Between Cefiderocol and Vancomycin

Drug interaction testing between cefiderocol and vancomycin was determined using the E-test, as previously described [30]. Briefly, the bacteria were prepared as above. Cefiderocol and vancomycin MIC strips were placed on the Mueller–Hinton agar inoculated lawns in a cross formation, with a 90° angle at the intersection between the values at their respective MICs for the test organism [31]. Plates were incubated for 24 h at 37 °C. The FIC index was determined for each combination using the following equation: FIC Index = (MIC cefiderocol combination/MIC cefiderocol alone) + (MIC vancomycin combination/MIC vancomycin alone). Antibiotic combinations that produce a 4-fold reduction in the MIC compared with the MICs of agents alone are considered to be synergistic (FIC index ≤ 0.5). An FIC index of >0.5 to 1.0 range is considered to be additive. An FIC index from 1 to 4 is defined as indifferent, while one that is >4 is antagonistic (29). In this instance, indifferent refers to no interaction between the antibiotics.

### 4.4. Statistical Analysis

The susceptibilities of the isolates for each antibiotic were determined using Fisher’s exact test (GraphPad, https://www.graphpad.com/quickcalcs/contingency1/, accessed on 21 July 2024).

## Figures and Tables

**Table 1:** MIC_50_ and MIC_90_ of endophthalmitis isolates to cefiderocol, amikacin, and ceftazidime in µg/mL.

Group	N	Cefiderocol		Amikacin		Ceftazidime	
		MIC_50_	MIC_90_	MIC_50_	MIC_90_	MIC_50_	MIC_90_
*Pseudomonadales* ^a^	16	0.0094	0.5	4	7	1.5	2.5
*Enterobacterales* ^b^	21	0.016	0.19	4	8	0.19	1.5
Other Gram-negative ^c^	3	0.023	ND ^e^	3	ND ^e^	0.5	ND ^e^
*S. aureus*	18	48	256	2.5	8	24	256
CNS ^d^	20	24	198.4	1.5	3.1	10	33.6

^a^ *Pseudomonadales* include 14 *Pseudomonas* species and 2 *Moraxella* species. ^b^ *Enterobacterales* include 10 *Serratia* species, 3 *Proteus*, *Morganella*, and *Klebsiella* species, 1 *Escherichia coli*, and 1 *Pantoea agglomerans*. ^c^ Other Gram-negative bacteria include 2 *Stenotrophomonas maltophilia* and 1 *Ralstonia pickettii*. ^d^ CNS, coagulase negative staphylococci. ^e^ ND, not determined.

**Table 2:** Susceptibility of endophthalmitis isolates to cefiderocol, amikacin, and ceftazidime based on systemic CLSI breakpoints.

Group ^a^	Cefiderocol ^b^	Amikacin ^c^	Ceftazidime ^d^
*Pseudomonadales*	16/16 (100%)	16/16 (100%)	16/16 (100%)
*Enterobacterales*	21/21 (100%)	13/21 (62%)	20/21 (95%)
Other Gram-negative	2/2 ^e^ (100%)	N/A	N/A
*S. aureus*	N/A	N/A	N/A
CNS	N/A	N/A	N/A

^a^ The breakdown of bacterial groups is the same as for Table 1. ^b^ The CLSI susceptibility breakpoint for cefiderocol is ≤4 µg/mL for both *Pseudomonadales* and *Enterobacterales*. The CLSI susceptibility breakpoint for cefiderocol is ≤1 µg/mL for *Stenotrophomonas maltophilia*. ^c^ The CLSI susceptibility breakpoint for amikacin is ≤16 µg/mL for *Pseudomonadales* and ≤4 µg/mL for *Enterobacterales*. ^d^ The CLSI susceptibility breakpoint for ceftazidime is ≤8 µg/mL for *Pseudomonadales* and ≤4 µg/mL for *Enterobacterales*. ^e^ There is a CLSI susceptibility breakpoint for cefiderocol for *Stenotrophomonas maltophilia* but not for *Ralstonia pickettii.* N/A, not applicable (no CLSI susceptibility breakpoints exist).

**Table 3:** MICs of endophthalmitis isolates to cefiderocol and vancomycin alone and in combination.

Group	Cefiderocol ^a^	Vancomycin ^a^	Cefiderocol Combination ^b^	Vancomycin Combination ^b^	FIC Index ^c^
*P. aeruginosa* E52	0.047	>256	0.047	>256	2.0
*P. aeruginosa* E68	0.047	>256	0.047	>256	2.0
*S. aureus* E860	96	1.0	96	1.0	2.0
*S. epidermidis* E878	8	1.0	4	0.75	1.25
CNS E879	12	3.0	8	2.0	1.33

^a^ MICs given as µg/mL. ^b^ Combination indicates synergy testing of cefiderocol and vancomycin; the higher value from duplicate experiments is shown. ^c^ FIC index—synergistic ≤ 0.5; additive > 0.5–1.0; indifferent 1.0–4.0; antagonistic > 4.0.

## Data Availability

Data are available upon request.

## References

[1] Chen A., Dun C., Schein O.D., Srikumaran D., Zafar S., Makary M., Woreta F. (2024). Endophthalmitis rates and risk factors following intraocular surgeries in the medicare population from 2016 to 2019. Br. J. Ophthalmol..

[2] Gurnani B., Kaur K. (2023). Endogenous Endophthalmitis. StatPearls.

[3] Samiy N., D’Amico D.J. (1996). Endogenous fungal endophthalmitis. Int. Ophthalmol. Clin..

[4] Jonas J.B., Knorr H.L., Budde W.M. (2000). Prognostic factors in ocular injuries caused by intraocular or retrobulbar foreign bodies. Ophthalmology.

[5] Simakurthy S., Tripathy K. (2023). Endophthalmitis. StatPearls.

[6] Jacobs D.J., Leng T., Flynn H.W., Shi W., Miller D., Gedde S.J. (2011). Delayed-onset bleb-associated endophthalmitis: Presentation and outcome by culture result. Clin. Ophthalmol..

[7] Gunalda J., Williams D., Koyfman A., Long B. (2023). High risk and low prevalence diseases: Endophthalmitis. Am. J. Emerg. Med..

[8] Schimel A.M., Miller D., Flynn H.W. (2013). Endophthalmitis isolates and antibiotic susceptibilities: A 10-year review of culture-proven cases. Am. J. Ophthalmol..

[9] Donahue S.P., Kowalski R.P., Eller A.W., DeVaro J.M., Jewart B.H. (1994). Empiric treatment of endophthalmitis. Are aminoglycosides necessary?. Arch. Ophthalmol..

[10] Gentile R.C., Shukla S., Shah M., Ritterband D.C., Engelbert M., Davis A., Hu D.-N. (2014). Microbiological spectrum and antibiotic sensitivity in endophthalmitis: A 25-year review. Ophthalmology.

[11] Stevenson L.J., Dawkins R.C.H., Sheorey H., McGuinness M.B., Hurley A.H., Allen P.J. (2020). Gram-negative endophthalmitis: A prospective study examining the microbiology, clinical associations and visual outcomes following infection. Clin. Exp. Ophthalmol..

[12] Mughal S., Sakina S.K. (2023). Artificial tears: Promising treatment or silent threat to public health?. Health Sci. Rep..

[13] Romanowski E.G., Mumper S.M., Shanks H.Q., Yates K.A., Mandell J.B., Zegans M.E., Shanks R.M.Q. (2024). Cefiderocol is an effective topical monotherapy for experimental extensively drug-resistant *Pseudomonas aeruginosa* keratitis. Ophthalmol. Sci..

[14] Centers for Disease Control and Prevention https://wwwn.cdc.gov/ARIsolateBank/Panel/IsolateDetail?IsolateID=10561&PanelID=10.

[15] Wang C., Yang D., Wang Y., Wentao N. (2022). Cefiderocol for the treatment of multidrug-resistant gram-negative bacteria: A systematic review of currently available evidence. Front. Pharmacol..

[16] McCreary E.K., Heil E.L., Tamma P.D. (2021). New Perspectives on Antimicrobial Agents: Cefiderocol. Antimicrob. Agents Chemother..

[17] Tillotson G.S. (2016). Trojan Horse Antibiotics-A Novel Way to Circumvent Gram-Negative Bacterial Resistance?. Infect. Dis. (Auckl.).

[18] Teran N., Egge S.L., Phe K., Baptista R.P., Tam V.H., Miller W.R. (2024). The emergence of cefiderocol resistance in *Pseudomonas aeruginosa* from a heteroresistant isolate during prolonged therapy. Antimicrob. Agents Chemother..

[19] Cassat J.E., Skaar E.P. (2013). Iron in infection and immunity. Cell Host Microbe.

[20] Kowalski R.P., Nayyar S.V., Romanowski E.G., Shanks R.M.Q., Mammen A., Dhaliwal D.K., Jhanji V. (2020). The prevalence of bacteria, fungi, viruses, and Acanthamoeba from 3,004 cases of keratitis, endophthalmitis, and conjunctivitis. Eye Contact Lens.

[21] Ito A., Sato T., Ota M., Takemura M., Nishikawa T., Toba S., Kohira N., Miyagawa S., Ishibashi N., Matsumoto S. (2017). In vitro antibacterial properties of cefiderocol, a novel siderophore cephalosporin, against Gram-negative bacteria. Antimicrob. Agents Chemother..

[22] Viale P., Sandrock C.E., Ramirez P., Rossolini G.M., Lodise T.P. (2023). Treatment of critically ill patients with cefiderocol for infections caused by multidrug-resistant pathogens: Review of the evidence. Ann. Intensive Care.

[23] Campochiaro P.A., Conway B.P. (1991). Aminoglycoside toxicity—A survey of retinal specialists. Implications for ocular use. Arch. Ophthalmol..

[24] Williams B.K., Joel Welch R., Nwanyanwu K.H., Shields C.L. (2019). Hemorrhagic occlusive retinal vasculitis leading to the diagnosis of ciliary body melanoma. Saudi J. Ophthalmol..

[25] Witkin A.J., Jaffe G.J., Srivastava S.K., Davis J.L., Kim J.E. (2023). Retinal Vasculitis After Intravitreal Pegcetacoplan: Report From the ASRS Research and Safety in Therapeutics (ReST) Committee. J. Vitreoretin. Dis..

[26] Simner P.J., Beisken S., Bergman Y., Ante M., Posch A.E., Tamma P.D. (2022). Defining Baseline Mechanisms of Cefiderocol Resistance in the Enterobacterales. Microb. Drug Resist..

[27] Kawai A., McElheny C.L., Iovleva A., Kline E.G., Sluis-Cremer N., Shields R.K., Doi Y. (2020). Structural basis of reduced susceptibility to Ceftazidime-Avibactam and Cefiderocol in *Enterobacter cloacaem* due to AmpC R2 loop deletion. Antimicrob. Agents Chemother..

[28] Romanowski J.E., Nayyar S.V., Romanowski E.G., Jhanji V., Shanks R.M.Q., Kowalski R.P. (2021). Speciation and antibiotic susceptibilities of coagulase negative Staphylococci isolated from ocular infections. Antibiotics.

[29] Harshaw N.S., Stella N.A., Lehner K.M., Romanowski E.G., Kowalski R.P., Shanks R.M.Q. (2021). Antibiotics used in empiric treatment of ocular infections trigger the bacterial Rcs stress response system independent of antibiotic susceptibility. Antibiotics.

[30] White R.L., Burgess D.S., Manduru M., Bosso J.A. (1996). Comparison of three different in vitro methods of detecting synergy: Time-kill, checkerboard, and E test. Antimicrob. Agents Chemother..

[31] Saiman L. (2007). Clinical utility of synergy testing for multidrug-resistant *Pseudomonas aeruginosa* isolated from patients with cystic fibrosis: ‘The motion for’. Paediatr. Respir. Rev..

